# Ultrasound imaging of the posterior lateral corner of the knee: a pictorial review of anatomy and pathologies

**DOI:** 10.1186/s13244-024-01606-x

**Published:** 2024-02-09

**Authors:** Wei-Ting Wu, Kentaro Onishi, Kamal Mezian, Ondřej Naňka, Bow Wang, Daniel Chiung-Jui Su, Vincenzo Ricci, Ke-Vin Chang, Levent Özçakar

**Affiliations:** 1 Department of Physical Medicine and Rehabilitation, National Taiwan University Hospital, Bei-Hu Branch, No. 87, Nei-Jiang Rd., Wan-Hwa District, Taipei, Taiwan; 2 Department of Physical Medicine and Rehabilitation, National Taiwan University College of Medicine, Taipei, Taiwan; 3 Department of Physical Medicine and Rehabilitation, University of Pittsburgh, Pittsburgh, PA USA; 4 Department of Orthopedic Surgery, University of Pittsburgh, Pittsburgh, PA USA; 5 Department of Rehabilitation Medicine, First Faculty of Medicine and General University Hospital in Prague, Charles University, Prague, Czech Republic; 6 Institute of Anatomy, First Faculty of Medicine, Charles University, Prague, Czech Republic; 7 Department of Medical Imaging, National Cheng Kung University Hospital, College of Medicine, National Cheng Kung University, Tainan, Taiwan; 8 Department of Physical Medicine and Rehabilitation, Chi-Mei Medical Center, Tainan, Taiwan; 9 Physical and Rehabilitation Medicine Unit, Luigi Sacco University Hospital, ASST Fatebenefratelli-Sacco, Milan, Italy; 10 Center for Regional Anesthesia and Pain Medicine, Wang-Fang Hospital, Taipei Medical University, Taipei, Taiwan; 11 Department of Physical and Rehabilitation Medicine, Hacettepe University Medical School, Ankara, Turkey

**Keywords:** Knee, Pain, Sonography, Sports injury, Ligament

## Abstract

Assessment of the posterior lateral knee pain poses diagnostic challenges, requiring accurate evaluation of various structures in light of the medical history and physical examination. Despite substantial progress in the ultrasonographic diagnosis of musculoskeletal disorders, the current protocol (EURO-MUSCULUS/USPRM. Basic scanning protocols for knee) fails to conduct a comprehensive investigation into the intricate, tendons, and ligaments of the posterior lateral knee. This pictorial review aims to bridge this gap by offering a systematic approach to utilize ultrasound examination of the less-discussed structures in this specific region. Providing cadaveric and magnetic resonance images, this essay demonstrates the efficacy of ultrasound in diagnosing posterior lateral knee pain. Notably, pathologies such as ligamentous sprains and tears are clearly discernible. Moreover, the integration of ultrasound guidance plays a vital role in reducing the risk of inadvertent neurovascular injury during injection, such as the common peroneal nerve and lateral genicular artery. This comprehensive approach will enhance clinicians’ understanding and equip them with effective management strategies for posterior lateral knee pain.

**Critical relevance statement** In this visual exposition, we delve into the intricacies of the posterior lateral corner of the knee. Offering a methodical approach to employ ultrasound for visualization of the less-explored structures within this region, the authors aim to enhance the diagnosis in posterior lateral knee pain.

**Key points**

1. Ultrasound excels at revealing intricate structures in the posterior lateral knee.

2. Due to proximity of vital structures, extreme caution is crucial during injections.

3. Employing dynamic scan and understanding ligaments enables a comprehensive exploration of pathologies.

**Graphical Abstract:**

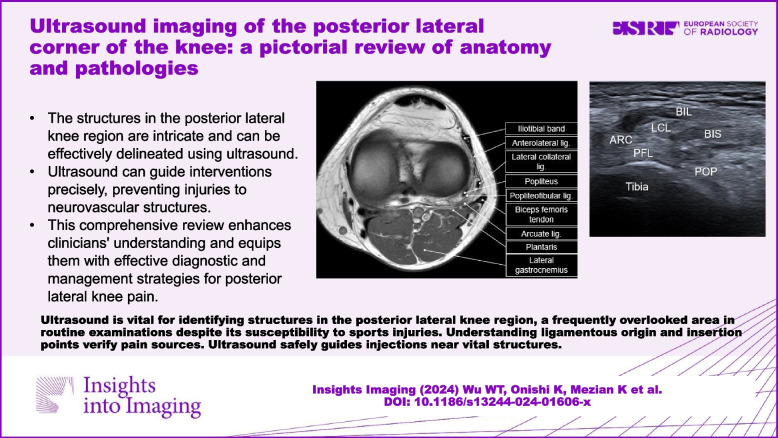

**Supplementary Information:**

The online version contains supplementary material available at 10.1186/s13244-024-01606-x.

## Introduction

Posterior lateral knee pain poses a diagnostic challenge whereby the clinical decision heavily relies on patient’s symptoms and physical examination. Various specific tests, such as the varus stress test (for integrity of the lateral collateral ligament), or Noble compression test (for iliotibial band friction syndrome) are utilized in this sense [1, 2]. Magnetic resonance (MR) imaging of the knee offers comprehensive insights into the structures within the posterolateral corner, facilitating the assessment of potential injuries. Variability in certain components of the posterolateral corner is observable on MR imaging with anatomic correlation [3]. However, concerns regarding cost and portability pose significant considerations when utilizing MR imaging for screening posterior lateral corner injuries.

On the other hand, ultrasound imaging has revolutionized the diagnosis of musculoskeletal disorders, including the painful knee [4, 5], owing to its zero radiation and superior resolution. However, the current scanning protocol (EURO-MUSCULUS/USPRM. Basic scanning protocols for knee) does not thoroughly investigate the complex fascia, tendon, and ligamentous systems at the posterior lateral knee [4]. Moreover, important but infrequent conditions such as popliteus myotendinous junction strain can be missed. Most of the existing articles present normal sonoanatomy without demonstrating the usefulness of ultrasound examination in delineating the relevant pathologies [6, 7]. In this pictorial essay, aiming to optimize prompt management, we revisit the anatomy of the posterior lateral corner of the knee and present a systematic ultrasound approach to image various less-discussed structures in this area. Cadaveric as well as MR images will also be added for providing a comprehensive overview of the posterior lateral corner of the knee.

## Anatomy

### Lamination of the posterior lateral knee

**Fig. 1:**
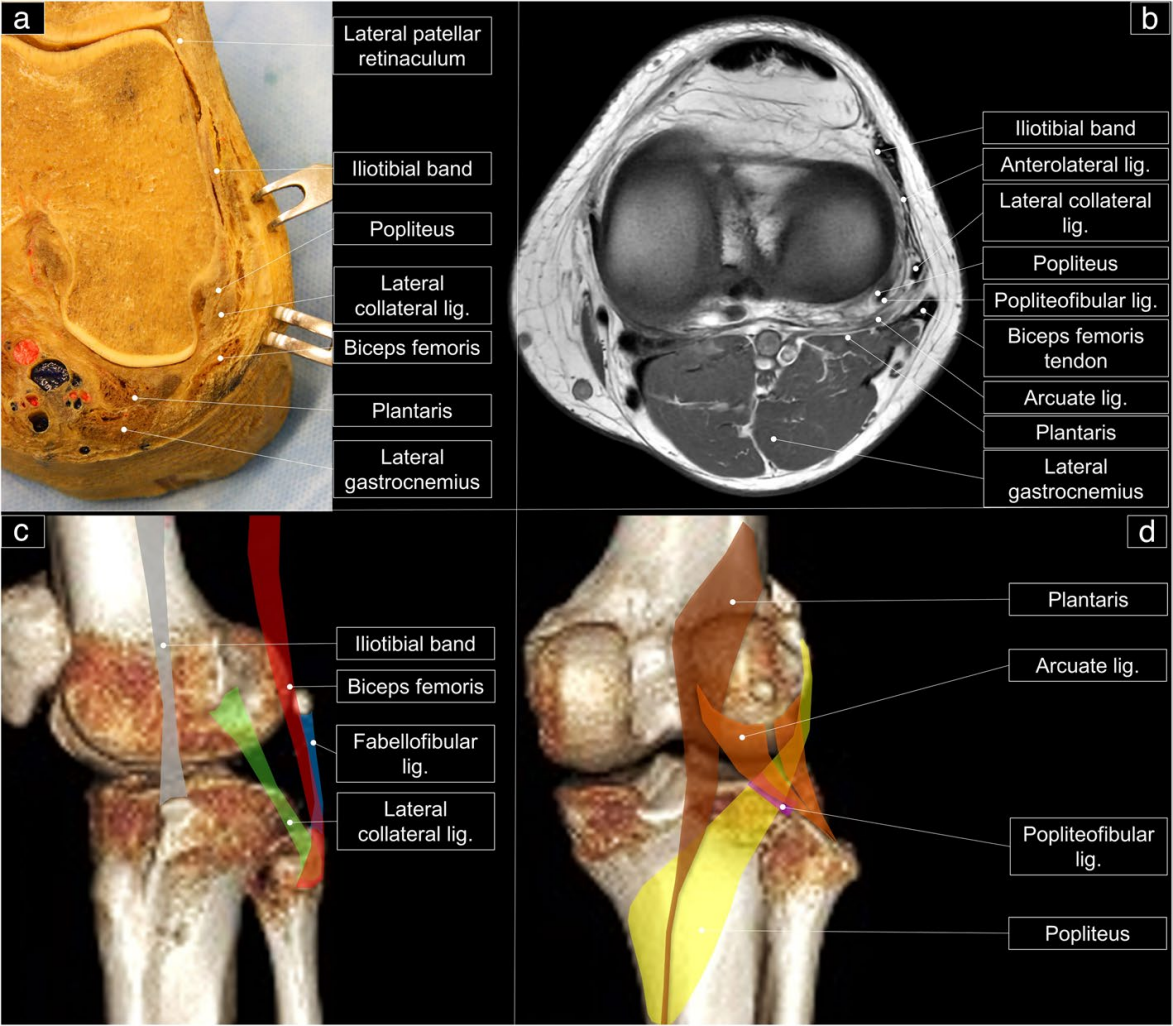
Cadaveric model (**a**) and magnetic resonance imaging (**a**) shows the posterior lateral corner of the knee in the axial plane. The schematic drawing elaborates the posterior lateral corner of the knee in sagittal (**c**) and coronal planes (**d**). Iliotibial band: grey shade; biceps femoris tendon, red shade; lateral collateral ligament: green shade; fabellofibular ligament: blue shade; arcuate ligament: orange shade; plantaris: brown shade; popliteus: yellow shade; popliteofibular ligament: purple shade

There are three layers in the posterior lateral knee (Fig. 1a and b) [8]. The first one comprises the iliotibial band anteriorly and the biceps femoris tendon posteriorly. The second layer consists of the lateral patellar retinaculum anteriorly and the lateral collateral ligament (LCL) posteriorly. The third is the capsular layer, which includes the fabellofibular and arcuate ligaments, with the popliteus tendon situated beneath it.

### Lateral collateral ligament

**Fig. 2:**
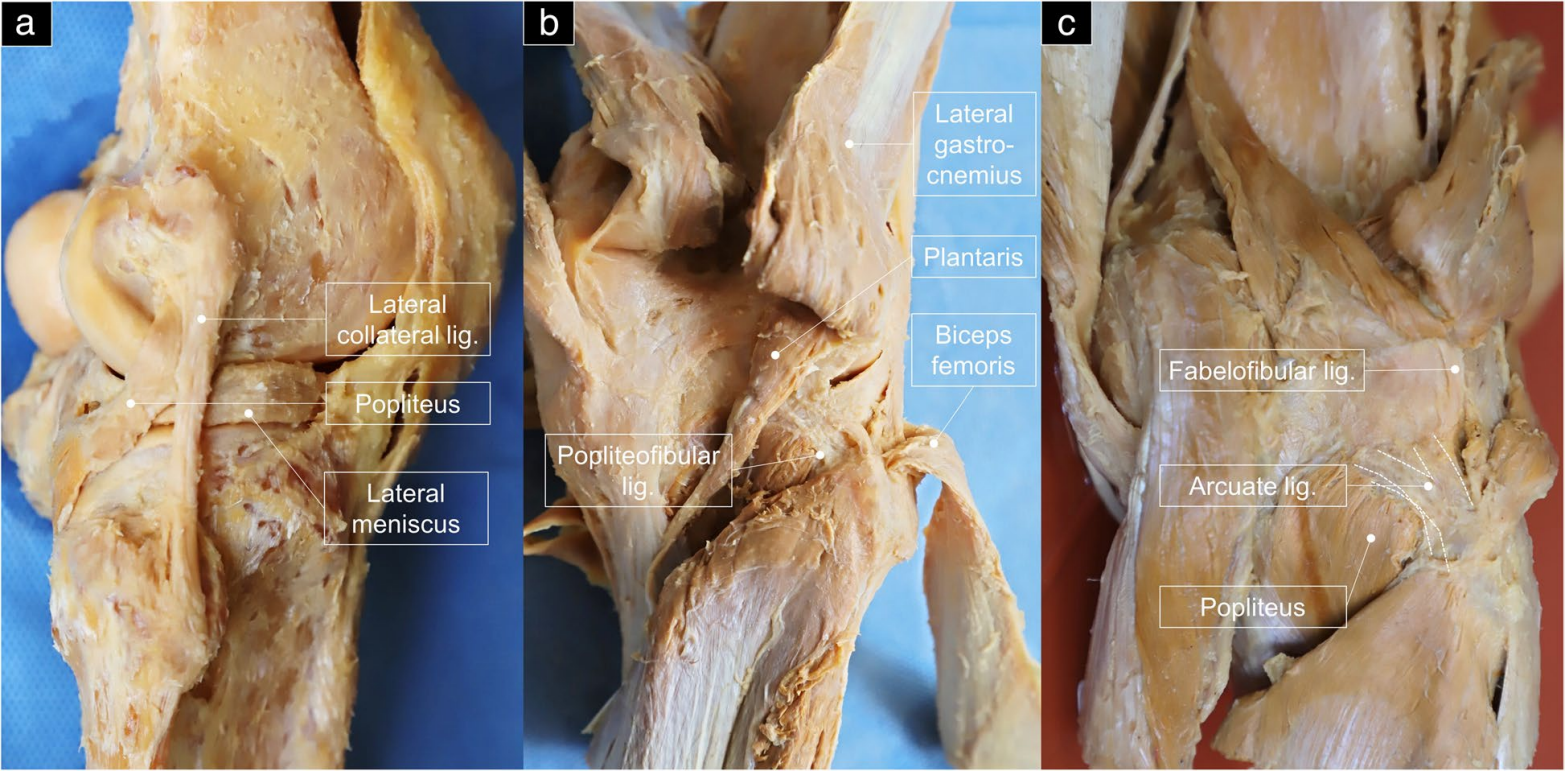
Cadaveric model shows the posterior lateral corner of the knee in sagittal plane (**a**) and coronal plane from the superficial (**b**) to the deep part (**c**)

**Fig. 3:**
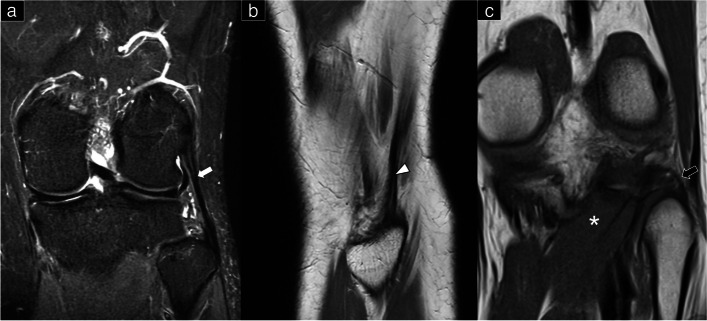
Magnetic resonance imaging shows the lateral collateral ligament in the coronal plane (**a**), biceps femoris tendon in the sagittal plane (**b**), and arcuate ligament in the coronal plane (**c**). White arrow: lateral collateral ligament; white arrowhead: biceps femoris tendon; black arrow: arcuate ligament; asterisk: popliteus

This ligament is an extra-articular structure that emerges from the external tuberosity of the lateral femoral condyle and fastens onto the fibular head (Figs. 1c, 2a, and 3a and Supplemental Fig. 1). Its cranial end sits atop the popliteal tendon and is a solitary, rounded bundle, resembling a pencil-like cord. Its fibers are completely independent of the knee joint capsule. The LCL inserts into a groove located at the lateral edge of the fibular head. A portion of its fibers proceeds distally, medial to the anterior arm of the long head of the biceps muscle, strengthening the fascia over the peroneus longus muscle. Notably, a bursa exists between the biceps femoris tendon and the LCL, forming an inverted “J” shape around the lateral, anterior, and anteromedial portions of the ligament [9]. The LCL plays a crucial role in restraining primary varus rotation, in addition to cooperating with the adjacent posterolateral structures during axial rotation of the tibia. As the knee flexes, its length is significantly reduced, becoming slackened.

### Biceps femoris tendon

This muscle (Figs. 1c, 2b, and 3b) has two (short and long) heads. The former arises from the posterior aspect of the femur, while the latter originates from the ischial tuberosity. Both heads have anterior and direct distal tendinous arms that insert into various structures, such as the lateral tibia plateau and the fibular head. The biceps femoris muscle is crucial in tibial external rotation and contributes to the knee’s forceful stability.

Using MR imaging, Shin et al. [10] examined the attachment pattern between the distal biceps tendon and the LCL. They identified five types as follows:

1. Type I: The LCL passes between the anterior arm and the direct arm of the long head of the biceps femoris tendon.
2. Type II: The LCL joins with the anterior arm of the long head of the biceps femoris tendon.
3. Type III: The biceps femoris tendon and the LCL join to form a conjoined tendon.
4. Type IV: The LCL passes laterally around the anterior margin of the biceps femoris tendon.
5. Type V: The LCL passes posterior to the direct arm of the long head of the biceps femoris tendon.

### Arcuate ligament

Extending from the apex of the fibular head, this ligament appears as Y-shaped and consists of a thin layer of connective tissue (Figs. 1d, 2c, and 3c). It courses over the musculotendinous junction of the popliteus before dividing into medial and lateral limbs. The medial one runs superomedially and attaches to the posterior knee capsule, while the lateral one attaches to the lateral femoral condyle and is stronger than the medial one. The lateral limb runs vertically, in close proximity to the lateral capsule. MR imaging studies report a wide range of its visibility, i.e., 24–87% [8, 11].

### Popliteofibular ligament

**Fig. 4:**
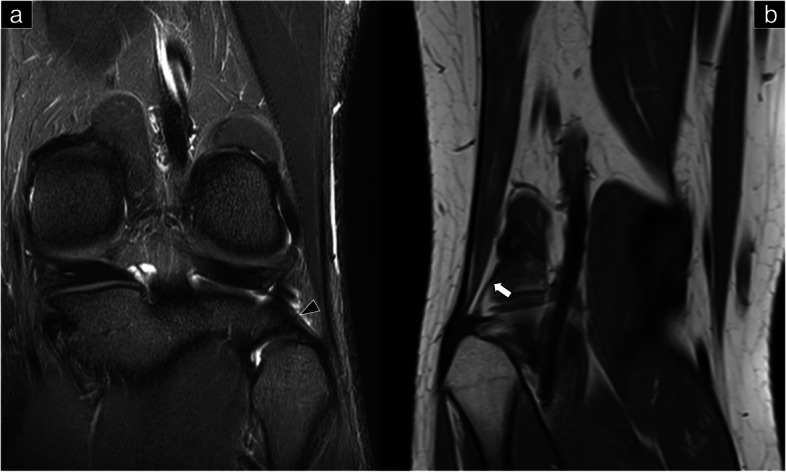
Magnetic resonance imaging shows the popliteofibular ligament (**a**) and the fabellofibular ligament in the coronal plane (**b**). Black arrowhead: popliteofibular ligament; white arrow: fabellofibular ligament

This ligament is a critical stabilizer of the knee joint and is located in the deepest layer of the posterolateral corner (Figs. 1d, 2b, and 4a). Along with the popliteus muscle, it plays an important role in preventing external tibial rotation, posterior translation, and varus angulation. In a cadaveric dissection study, the popliteofibular ligament was identified in all specimens (100%) [12]. Originating from the medial popliteal tendon just proximal to the myotendinous junction, the popliteofibular ligament attaches to the fibular head, situated posteriorly to the LCL. The popliteofibular ligament lies deep to the arcuate ligament and the inferior lateral genicular artery.

### Fabellofibular ligament

This ligament serves as a static stabilizer and runs from the fibular head to the fabella (Figs. 1c, 2c, and 4b). The fabella is a sesamoid bone located within the lateral head of the gastrocnemius tendon. Its occurrence in humans is highly variable, i.e., 20 to 87% [13]. The ligament may be present even without the fabella whereby, in such cases, it originates from the posterolateral surface of the lateral femoral condyle. The inferior lateral genicular artery, a branch of the popliteal artery, passes laterally around the posterior joint capsule and runs anterior to the fabellofibular ligament. Interestingly, the sizes of the fabellofibular and arcuate ligaments appear to have an inverse relationship [6].

### Plantaris muscle

This muscle is situated in the posterior compartment of the leg, anterior to the lateral head of the gastrocnemius muscle and posterior to the popliteal muscle (Figs. 1a, b, d and 2b and Supplemental Fig. 1). Its origin is from the lateral supracondylar line of the femur and the posterior knee capsule. Its tendon courses distally between the medial gastrocnemius and the soleus muscles, before inserting into the calcaneus medial to the Achilles tendon. In a cadaveric study of 47 spontaneously aborted human fetuses, the plantaris muscle was present in 74 lower limbs (78.7%) and absent in 20 limbs (21.3%) [14]. While the muscle may be considered vestigial from an evolutionary perspective, its proximal part (muscle and tendon portions alike) is susceptible to various injuries.

### Popliteus tendon/muscle

The popliteus tendon is an extra-synovial structure that inserts into the popliteal groove of the femur and is strongly adherent to the joint capsule (Figs. 1d and 2a, c). Its femoral insertion is rarely injured whereas its myotendinous junction is more commonly affected [15]. The popliteal sulcus, located at the posterolateral aspect of the femoral condyle, aids in localizing the femoral insertion of the tendon. The popliteus (a flat and triangular-shaped) muscle is both intra-capsular and extra-synovial. It is a deep knee joint muscle, serving as the floor of the popliteal fossa and separating the lateral meniscus of the knee from the LCL. This muscle inserts on the tibia just proximal to the soleal line but below the tibial condyles, and it is supplied by the tibial nerve [16]. As the muscle contracts, it leads to flexion and lateral rotation of the femur on the tibia, unlocking the knee joint.

## Ultrasound imaging—normal

### Ultrasound imaging acquisition

In this pictorial review, normal ultrasound images were obtained from a 38-year-old healthy male participant (the first author) without complaints of knee pain and with no previous history of trauma, following an established scanning protocol [17]. The Institutional Review Board Statement was not applicable as the present article is a pictorial essay. We employed high-frequency (18 MHz) linear and curvilinear (9.7 MHz) transducers (HI VISION, Ascendus, Hitachi, Japan, and Aplio i600, Canon, Tokyo, Japan) to scan the target structures. The transducer was positioned in the short axis of tendons, ligaments, and muscles to outline their cross-sections and reciprocal positions with adjacent soft tissues. Simultaneously, the transducer was oriented along the long axis of the afore-mentioned structures to facilitate the prompt identification of the fibrillar configuration. Moreover, Doppler imaging was employed to discern vessels accompanying or peri/intraneural hypervascularity linked to inflammatory pathologies. Additionally, a checklist is included in the supplemental materials to provide detailed guidance on the transducer positioning for ultrasonographic scanning of the posterior lateral corner of the knee.

### Iliotibial band

During the examination, the patient adopts a lateral decubitus position, placing the target knee over the contralateral one, with the patient’s face turned toward the examiner. The transducer is positioned at the axial plane of the lateral femoral condyle and moved from anterior to posterior, visualizing the iliotibial band and the biceps femoris muscle, respectively (Supplemental Fig. 2A). These structures are linked by a thin connective tissue known as the anterior lateral complex of the knee (also know as the anterolateral ligament in some literature) [18], and it can also be observed in the same plane.

To scan the iliotibial band, the transducer can be placed at the axial plane and moved from the lateral femoral condyle to the proximal tibia. By pivoting the transducer along the coronal plane, the long axis of the iliotibial band can be visualized as a hyperechoic fibrillar band crossing over the lateral femoral condyle and inserting onto the Gerdy’s tubercle of the tibia (Supplemental Fig. 2B) [19]. This allows for a detailed assessment of the entire length of the iliotibial band at the knee joint level. The long axis of the anterolateral ligament (Supplemental Fig. 2C) and biceps femoris tendon (Supplemental Fig. 2D) can be visualized as a hyperechoic fibrillar band crossing over the lateral femoral condyle and inserting onto the tibia and fibula, respectively.

### Lateral collateral ligament

**Fig. 5:**
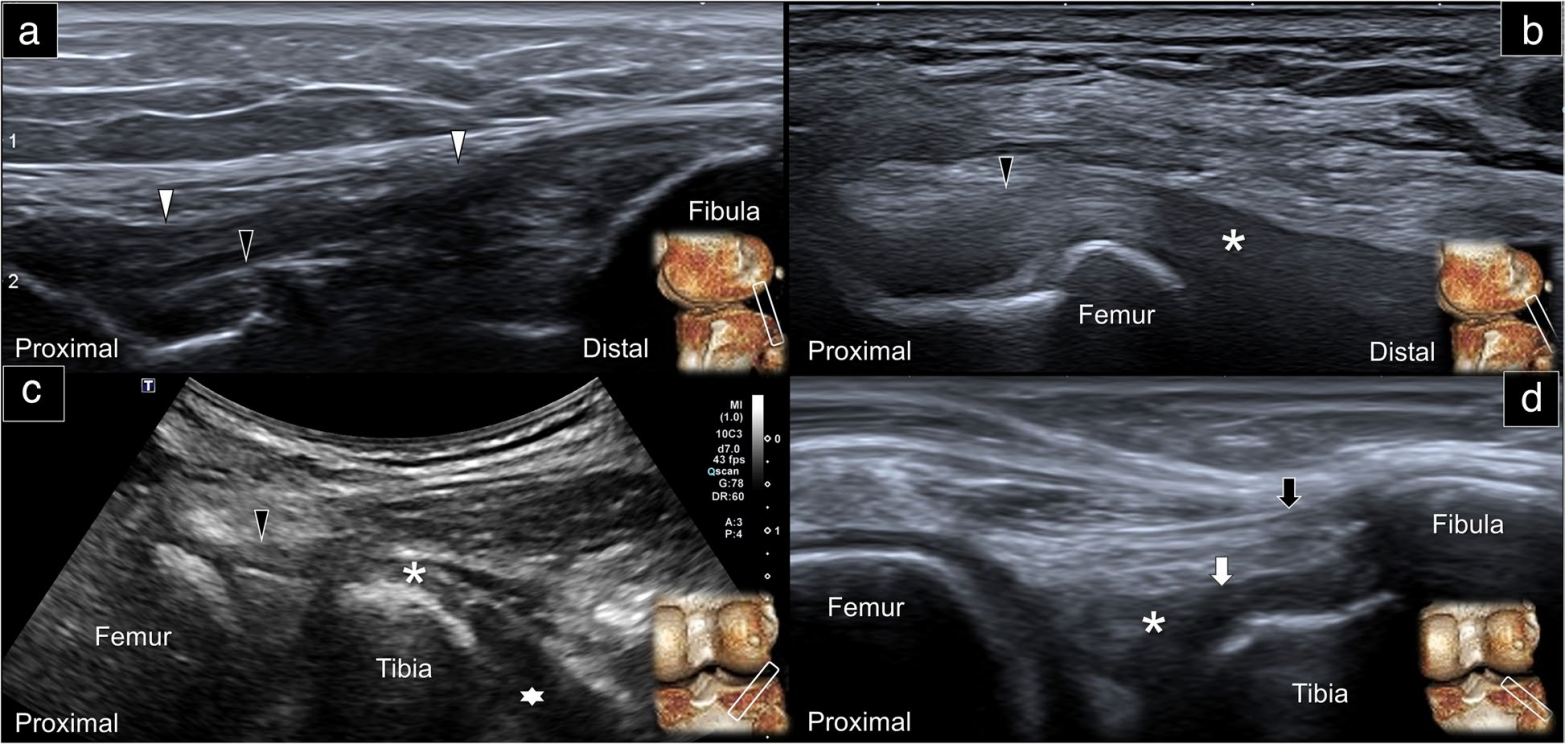
Sonographic imaging of the lateral collateral ligament, popliteus tendon (**a**), popliteus myotendinous junction (**b**), popliteus muscle (**c**), and popliteofibular and arcuate ligaments (**d**) using the long-axis approach. White arrowhead: lateral collateral ligament; black arrowhead: popliteus tendon; asterisk: popliteus myotendinous junction; star: popliteus muscle; white arrow: popliteofibular ligament; black arrow: arcuate ligament; white rectangles: transducer positions

Returning to the level of the lateral femoral condyle, the short axis of the LCL can be visualized beneath the anterolateral ligament and anterior to the biceps femoris muscle (Supplemental Fig. 2A). As the transducer is moved distally to cross the lateral meniscus, the attachment of the LCL to the fibular head becomes visible. By pivoting the transducer in the oblique coronal plane, the long axis of the LCL can be visualized (Fig. 5a), providing a more comprehensive evaluation of the ligament.

### Popliteus tendon/muscle

When imaging the knee on the coronal plane, the oblique axis of the popliteus tendon can be observed beneath the long-axis of the LCL (Fig. 5a). To visualize the myotendinous junction of the popliteus (Fig. 5b), the distal end of the transducer should be rotated slightly more posteriorly, while the proximal end is fixed on the popliteal sulcus of the lateral femoral condyle. Since the popliteus tendon and popliteal muscle do not align in a straight line, the transducer needs to be moved further distally with its distal end rotated more horizontally to identify the long axis of the popliteus muscle on the posterior proximal tibia (Fig. 5c).

### Popliteofibular ligament

Moving back to the long-axis imaging of the popliteus myotendinous junction, a hyperechoic band-like structure is seen emerging from the side, coursing on top of the proximal posterior lateral tibia and attaching to the fibular head. This structure is the popliteofibular ligament (Fig. 5d).

### Arcuate ligament

The arcuate ligament (Fig. 5d), another thin hyperechoic structure, can be observed coursing on top of the popliteofibular ligament. While its distal attachment is also the fibular head, it is situated more superficial than the latter. Unlike the popliteofibular ligament, which attaches to the side of the popliteus myotendinous junction, the arcuate ligament is observed passing over the popliteus myotendinous junction and inserting on the posterior lateral femoral condyle. To visualize this insertion point, the transducer should be moved more proximally and anteriorly, until it is parallel to the long axis of the biceps femoris tendon.

**Fig. 6:**
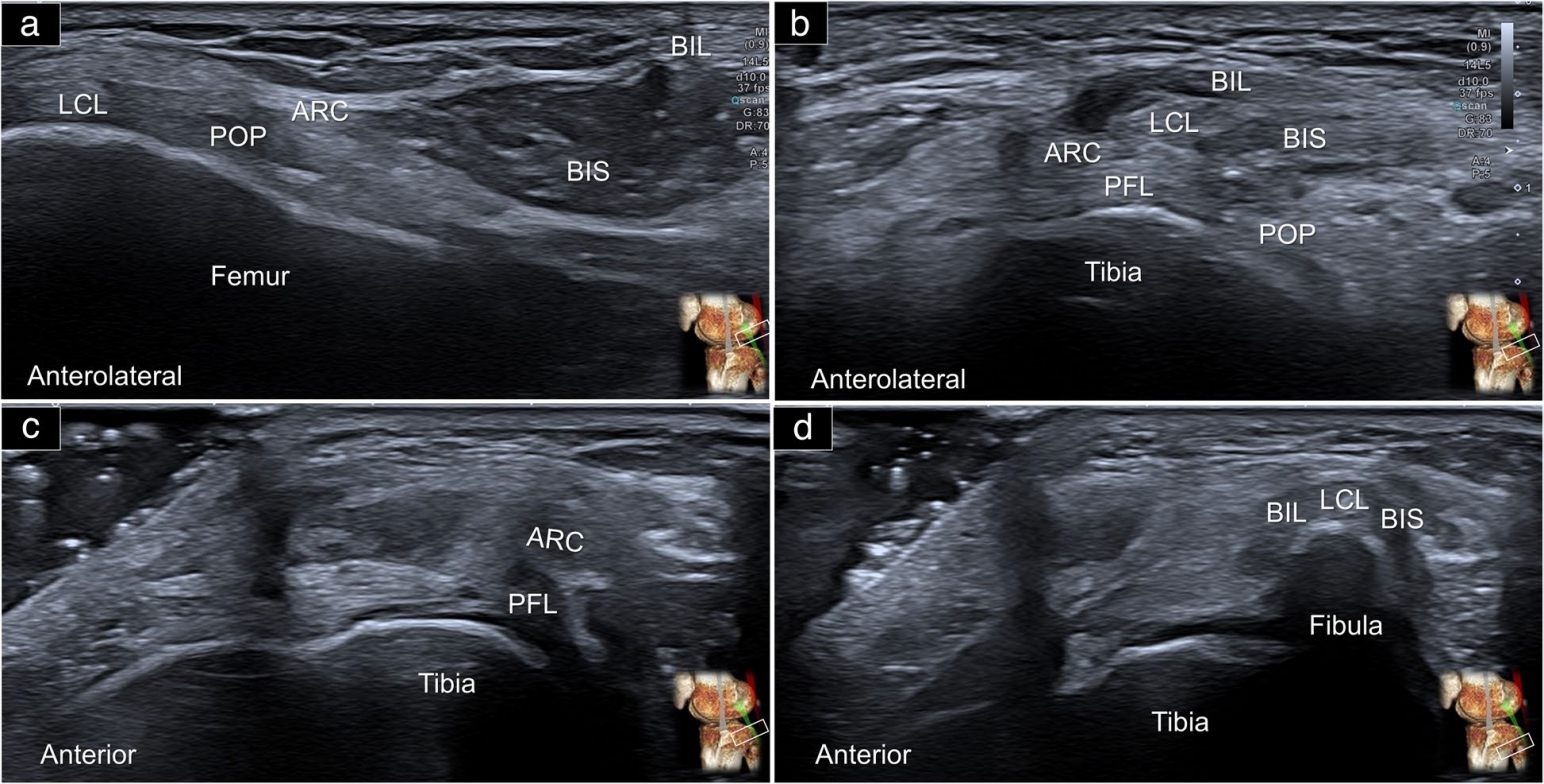
Sonographic imaging displays the posterior lateral corner of the knee in the transverse plane, at the level of the lateral femoral condyle (**a**), across the tibial plateau (**b**), over the proximal tibia shaft (**c**), and at the fibular head (**d**). This comprehensive view facilitates the visualization of both the arcuate and popliteofibular ligaments. LCL: lateral collateral ligament; BIL: long head tendon of the biceps femoris; BIS: short head tendon of the biceps femoris; ARC: arcuate ligament; PFL: popliteofibular ligament

An alternative method of visualizing the arcuate and popliteofibular ligaments is by placing the transducer over the posterior lateral knee joint line in the axial plane. The biceps femoris tendon can be observed easily in the short-axis, underneath the subcutaneous tissue. Beneath the biceps femoris tendon is a layer of hypoechoic fat tissue. As the transducer is moved towards the fibular head, two hypoechoic circles sequentially appear deep to the fat tissue. The first one appearing on the surface corresponds to the arcuate ligament, while the second deeper circle corresponds to the popliteofibular ligament (Fig. 6, Supplemental Video 1).

### Biceps femoris tendon

The biceps femoris tendon can be scanned by starting in the axial plane, with the transducer placed over the lateral femoral condyle. Once its myotendinous junction is localized (Supplemental Fig. 3A), the transducer can be swept distally. The biceps femoris can be seen moving from posterior to anterior and then bifurcating to encircle the LCL, which resembles Pac-Man (Supplemental Fig. 3B). The upper lip of the Pac-Man originates from the long head of the biceps femoris, while the lower lip is derived from the short head. Before the biceps femoris finally attaches to the fibular head, an anterior bundle emerges from the short head to insert on the tibial plateau (Supplemental Video 2). The biceps femoris muscle eventually reaches the fibular head. Its long head is positioned in front of the attachment point of the LCL, while its short head is located behind the LCL’s attachment point (Supplemental Fig. 3C). The transducer can be pivoted to the coronal plane to visualize the distal biceps femoris tendon in the long-axis view (Supplemental Fig. 3D).

### Fabellofibular ligament

**Fig. 7:**
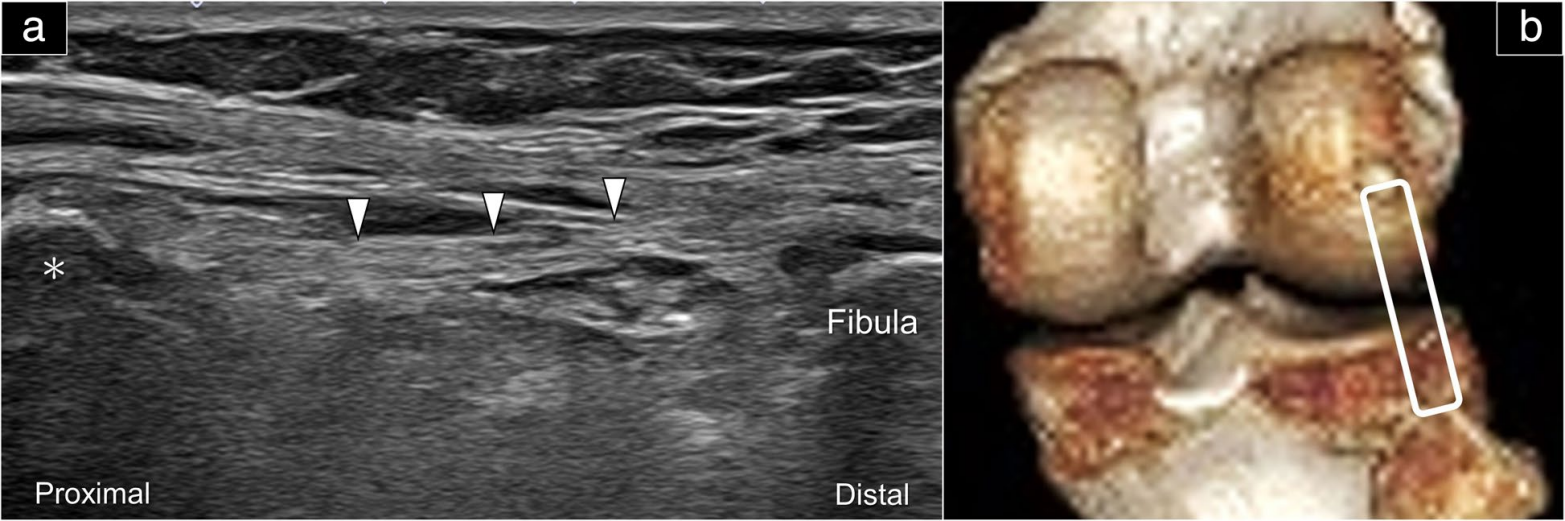
Sonographic imaging of the fabellofibular ligament in the long-axis view (**a**). The colored dashed rectangles indicate the transducer’s positions (**b**). White arrowhead: fabellofibular ligament; asterisk: fabella

The fabellofibular ligament can be easily observed when the fabella is present (Fig. 7). To identify the fabella, the transducer is placed in the axial plane over the posterior knee crease. It is then slowly rotated to the oblique sagittal plane until it reaches the fibular head. At this point, the fabellofibular ligament appears as a hyperechoic band composed of fine fibrils that connect the fabella and the fibular head.

### Plantaris muscle

Placing the transducer in the axial plane over the posterior femoral condyle, the plantaris muscle can be seen underneath the lateral gastrocnemius muscle (Supplemental Fig. 4A-C) [20, 21]. Moving the transducer more distally, the plantaris muscle gradually tapers and becomes a tendon coursing in the fascial plane between the medial gastrocnemius and soleus muscles (Supplemental Fig. 4D). Pivoting the transducer in the oblique sagittal plane, the whole tendon muscle complex is observed attaching to the posterolateral aspect of the lateral femoral condyle (Supplemental Fig. 4E and F).

## Ultrasound imaging—pathologies

When patients present with complaints of posterior lateral knee pain, the dial test can be employed to assess instability prior to the ultrasound evaluation [22]. The participant is positioned in a prone position with their knees flexed at 30°. The examiner grasps the patient’s heels and externally/maximally rotates the legs. Subsequently, the knees are flexed to 90° while applying maximal external rotation once more. A positive result is indicated by a discrepancy of ≥ 10° in the foot-thigh angle (unaffected vs. painful sides).

Following the physical examination, a systematic scan of the structures can be performed, as previously described. In a 2010 study [7] evaluating the effectiveness of dynamic ultrasound in identifying posterolateral corner knee injuries requiring surgical intervention, sixteen patients underwent both ultrasound and subsequent surgery. Of these, twelve patients displayed surgical findings necessitating intervention on the posterolateral knee structures. Assessment of static ultrasound images revealed the following results: for the LCL, sensitivity and specificity were 92% and 75%, respectively; for the popliteus, sensitivity and specificity were 33% and 100%, respectively; and for the popliteofibular ligament, sensitivity and specificity were 67% and 75%, respectively [7]. Furthermore, the dynamic ultrasound stress test, indicating a lateral joint space width of 10.5 mm or more during varus stress, demonstrated a sensitivity of 83% and a specificity of 100% in recognizing injuries of the posterolateral corner complex [7].

**Fig. 8:**
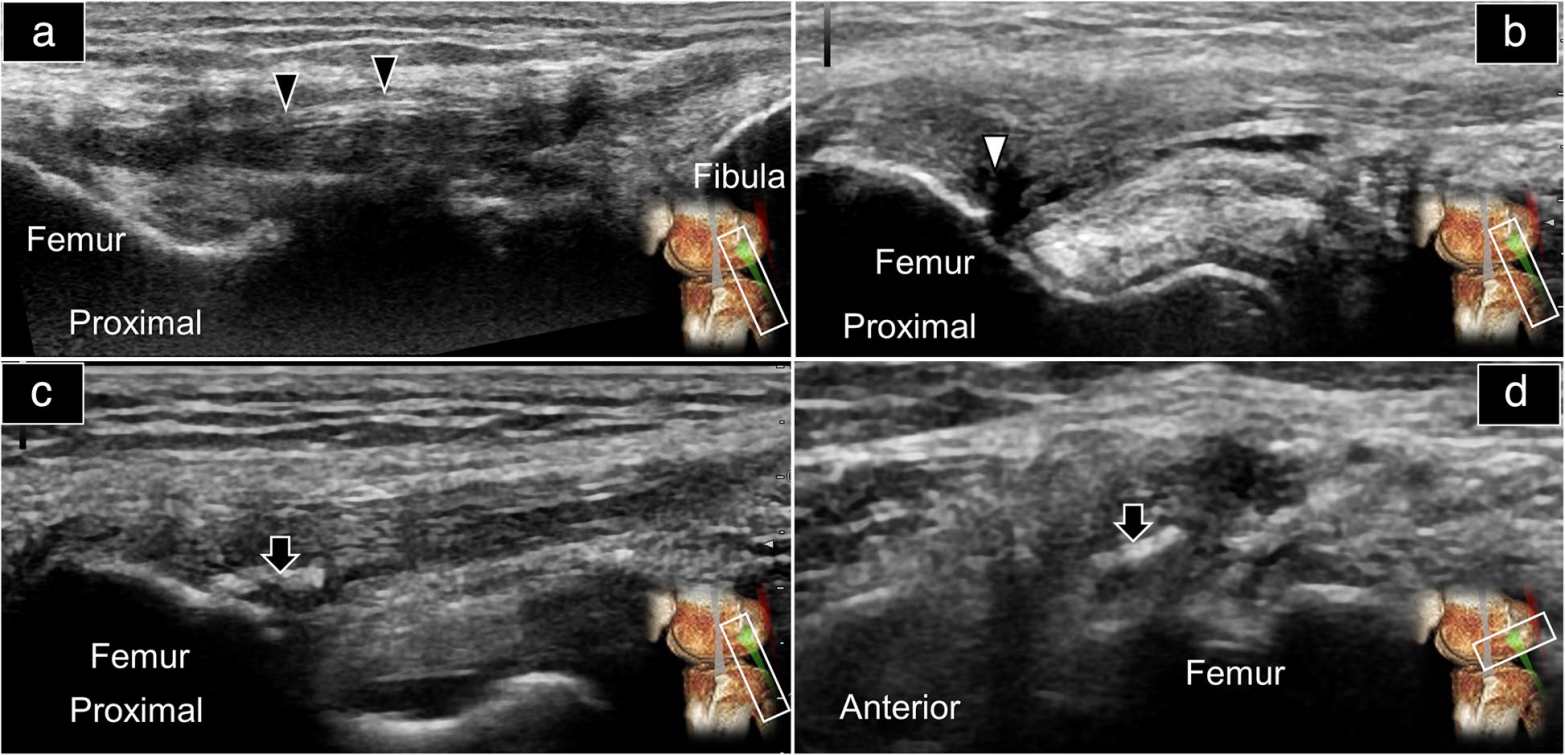
Ultrasound imaging using the long-axis approach revealed the thickened and hypoechoic changes (black arrowheads) (**a**), an anechoic region consistent with a partial thickness tear (white arrow) (**b**), and calcification (black arrows) in long-axis (**c**) and short-axis (**d**) views of the lateral collateral ligament. Please also reference Fig. 5A for the normal appearance of the lateral collateral ligament

In this pictorial review, pathological ultrasound images were obtained retrospectively from the image data registry. In the case of a sprained LCL (Fig. 8a, Supplemental Video 3), thickening and hypoechoic appearance may be observed, accompanied by a blurred fibrillary pattern. In partial tears (Fig. 8b), an intratendinous slip or a hypoechoic shadow may be visualized, typically located on the undersurface of the ligament, with some effusion present between the ligament and the interface with the popliteal tendon. Calcification at the ligament’s deep part may be seen in chronic cases (Fig. 8c and d). In complete tears, hematoma (besides retracted ligament fibers) may be apparent.

**Fig. 9:**
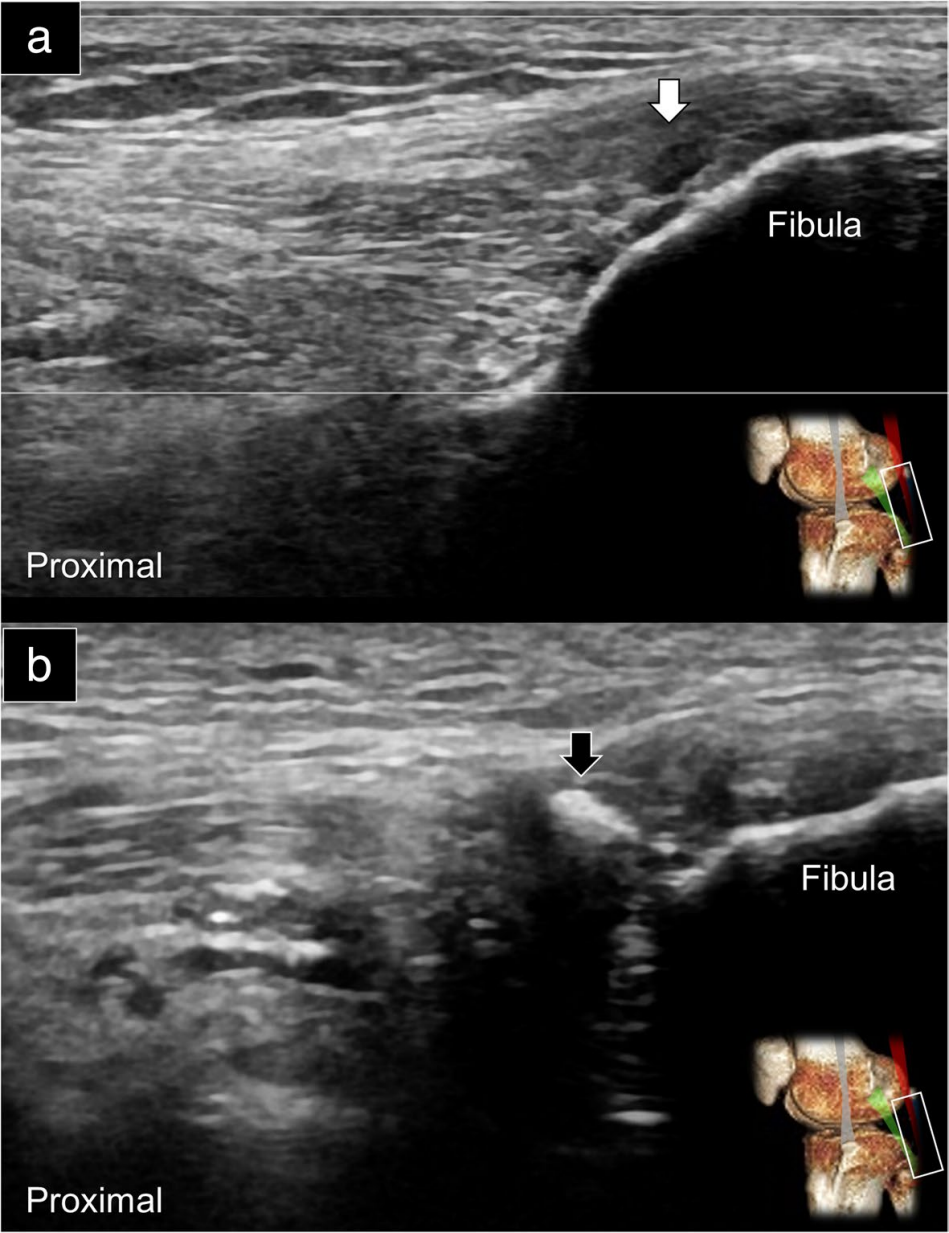
Ultrasound imaging using the long-axis approach revealed an anechoic region, which was consistent with a partial thickness tear (white arrow) of the biceps femoris tendon (**a**) and calcification inside its distal insertion (black arrow) (**b**). Please also reference Supplemental Fig. 2 for the normal appearance of the biceps femoris tendon

**Fig. 10:**
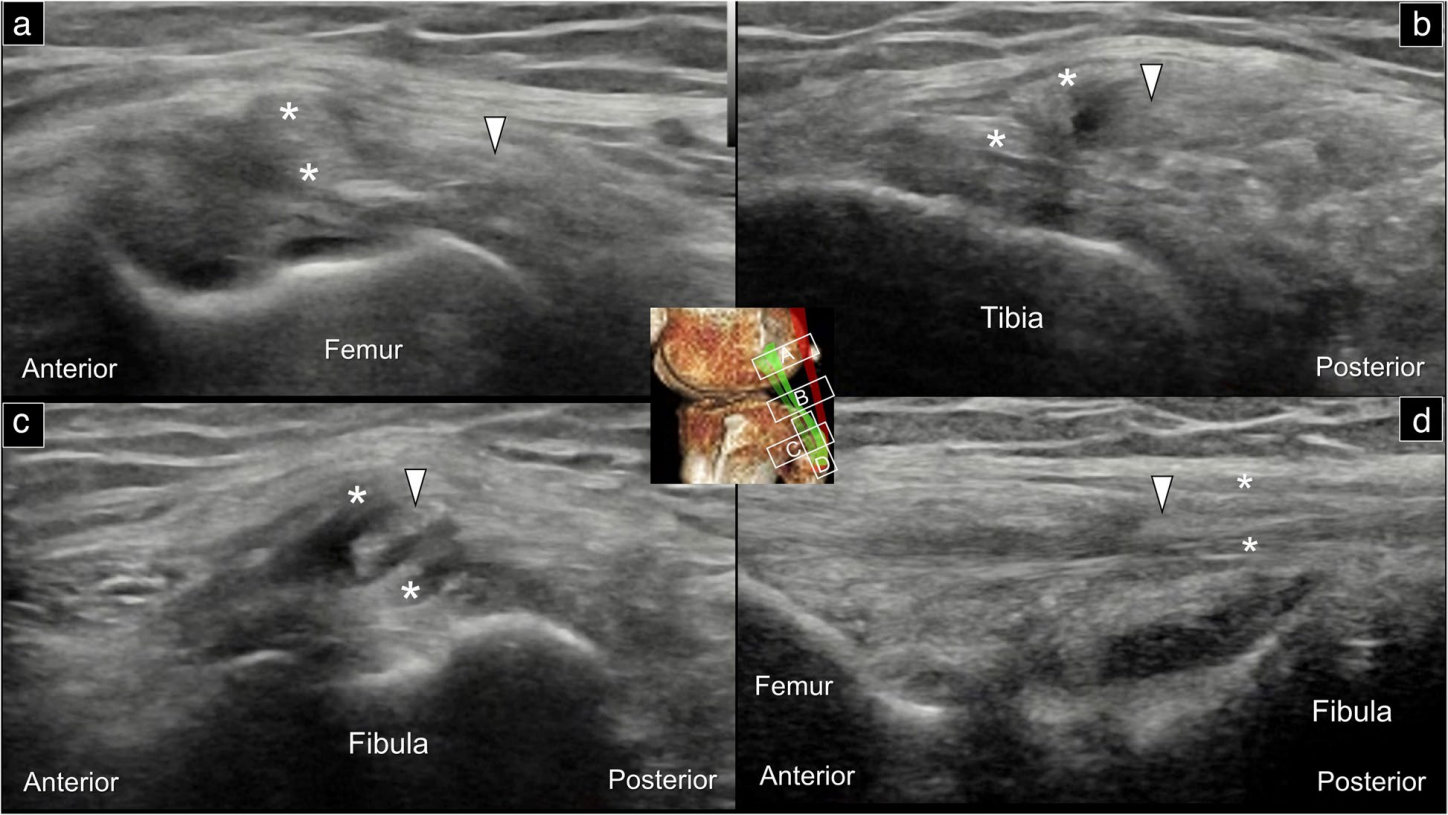
Ultrasound imaging of the bifid lateral collateral ligament (asterisk) from the origin (**a**) and middle part (**b**) to the insertion (**c**) in the short-axis and long-axis (**d**) views. White arrowheads: biceps femoris tendon. Please reference Supplemental Fig. 2 for the normal depiction of the biceps femoris tendon and its relationship with the lateral collateral ligament

Similar pathologies can be observed in the distal biceps femoris tendon. In cases of partial tears (Fig. 9a), a hypoechoic region may be detected in the lower half of the tendon’s attachment. In instances of previous complete tears, although the hematoma may have resolved, calcification can develop (Fig. 9b), accompanied by thickening and loss of the fibrillary appearance at the distal attachment site. Furthermore, it is worth noting that in most individuals, the distal biceps femoris tendon wraps around both the upper and lower halves of the LCL. However, certain normal variations can occur where a bifid LCL (coursing at the anterior aspect of the distal biceps femoris tendon) may envelop the tendon itself (Fig. 10, Supplemental Video 4).

**Fig. 11:**
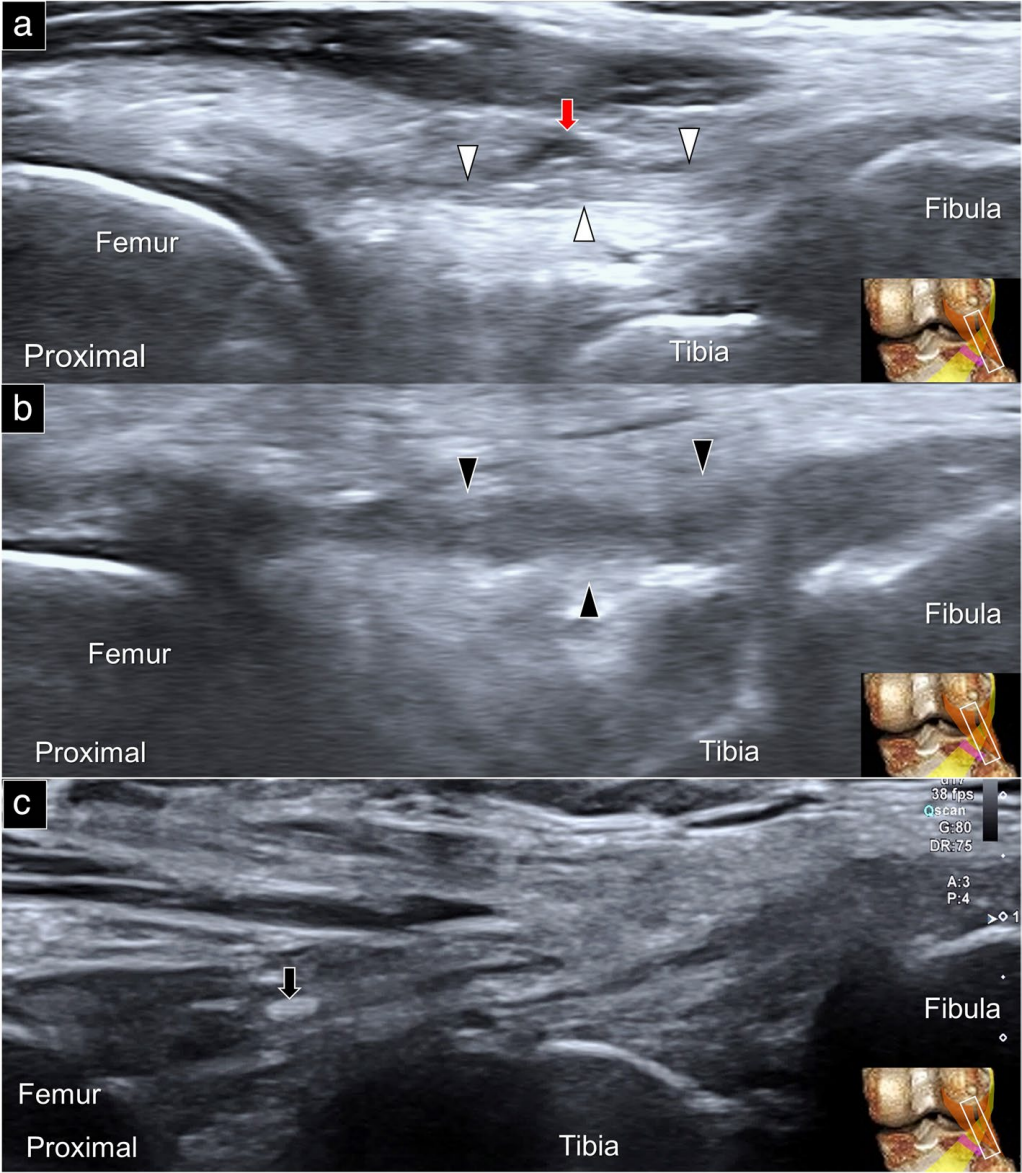
Sonographic imaging (long-axis view) depicts normal (white arrowheads) (**a**), thickened/hypoechoic (black arrowheads) (**b**), and calcified (black arrow) (**c**) arcuate ligaments. Note that the inferior lateral genicular artery (red arrow) is visible adjacent to the arcuate ligament

Arcuate ligament sprain is typically observed at its attachment to the fibula whereby intra-ligamentous calcification can be identified following severe lesions. A useful approach to confirm the presence of thickening and hypoechoic appearances on the affected side is to compare it with the asymptomatic/contralateral side (Fig. 11). Conducting dynamic scanning of the arcuate ligament in the short-axis view aids in distinguishing it from the underlying popliteofibular ligament (Supplemental Fig. 5, Supplemental Video 5). Additionally, the short-axis view allows for identification of the relationship between the arcuate ligament and the common peroneal nerve. By employing the same scanning plane utilized for the arcuate ligament, it is feasible to diagnose popliteofibular ligament pathologies. For instance, a sprain lesion can be determined by comparatively observing the augmented thickness and diminished echogenicity of the affected ligament (Supplemental Fig. 6).

**Fig. 12:**
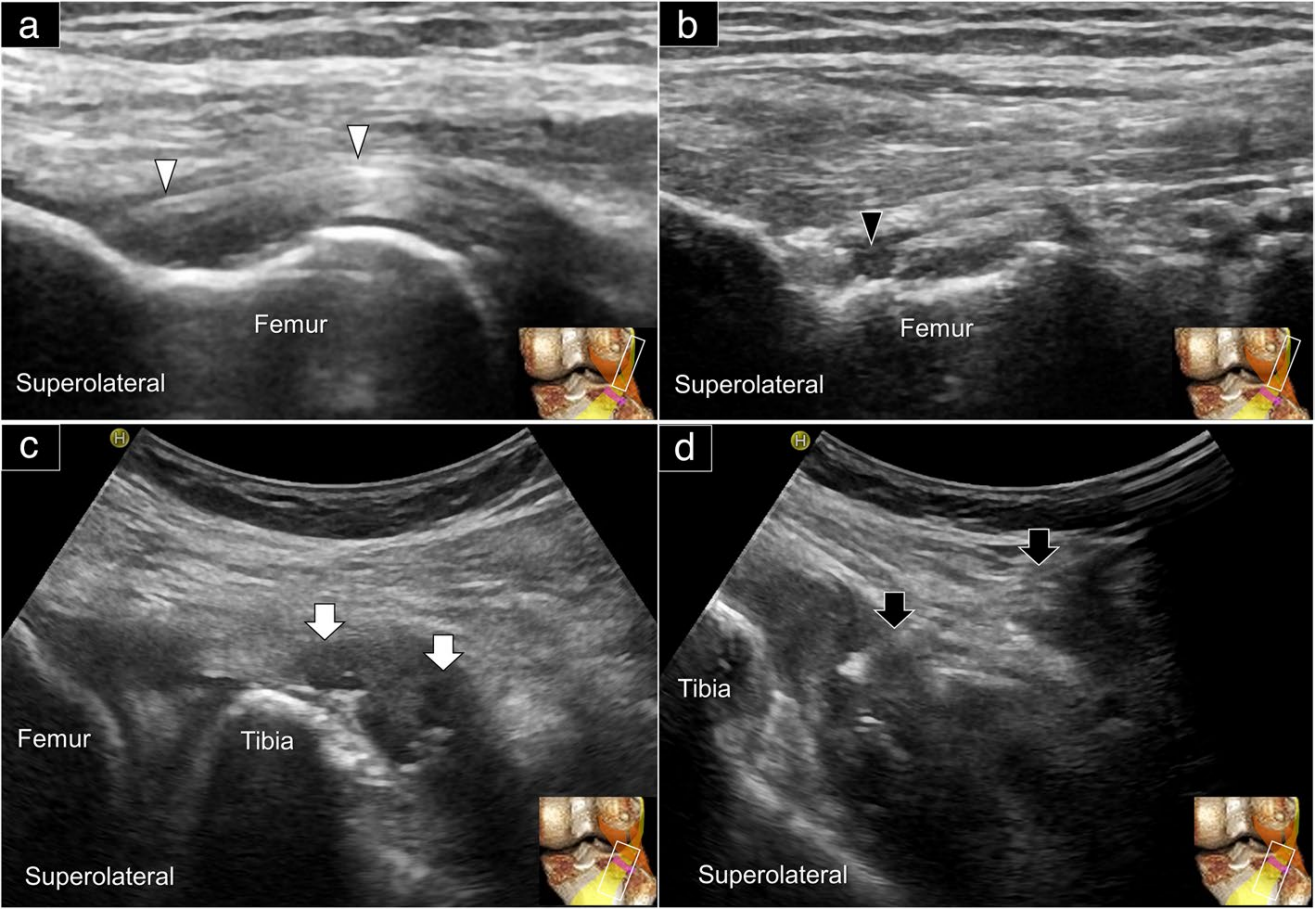
Ultrasound imaging (long-axis view) of the normal (white arrowhead) (**a**) and partially torn (black arrow) (**b**) popliteus tendons. Popliteus muscle tear (white arrow) (**c**) and the ultrasound-guided injection (**d**). Black arrow: needle trajectory

The popliteus tendon is situated in the deepest layer of the posterior lateral corner of the knee, making it challenging to pinpoint the exact location of symptoms resulting from its injury. When suspected as the source of pain, the scanning should encompass its tendon part, myotendinous junction and the muscle belly. Common types of popliteus tendon injuries include sprains and partial/complete tears (Fig. 12a and b). Notably, the injury might extend to the muscle portion whereby treatment might include ultrasound-guided regenerative injection (Fig. 12c and d). Another relevant condition would be a ganglion cyst (Supplemental Fig. 7, Supplemental Video 6), which can be effectively treated by ultrasound-guided aspiration, while taking precautions to avoid collateral damage to the common peroneal nerve [23].

## Conclusion

The posterior lateral corner of the knee is a frequently overlooked area in routine ultrasound examinations, despite its susceptibility to sports injuries. A comprehensive understanding of the regional anatomy helps investigators identify potential sources of pain, where systematic ultrasound examination can be invaluable for uncovering common pathologies including ligamentous sprains and tears. It is important to note that the posterior lateral corner is traversed by the common peroneal nerve and inferior lateral genicular artery, and ultrasound guidance can, for sure, minimize their risk of accidental injury effectively.

### Supplementary Information

**Additional file 1:** Checklist of the PLC of the Knee. **Supplemental Fig. 1.** Magnetic resonance imaging shows the posterior lateral corner of the knee in the axial plane at the tibiofibular joint level. **Supplemental Fig. 2.** Sonographic imaging shows the posterior lateral corner of the knee in the transverse plane (A). Corresponding ultrasound images (long-axis view) of the iliotibial band (B), the anterolateral ligament (C), and the biceps femoris tendon (D). ITB: iliotibial band; ALL: anterolateral ligament; LCL: lateral collateral ligament; LM: lateral meniscus; BIL: long head tendon of the biceps femoris; BIS: short head tendon of the biceps femoris. **Supplemental Fig. 3.** Sonographic imaging shows the biceps femoris in the transverse plane; the myotendinous junction (A), anterior bundle from the short head (B), its attachment to the fibular head (C). Imaging in long-axis view (D). LCL: lateral collateral ligament; BIL: long head tendon of the biceps femoris; BIS: short head tendon of the biceps femoris; LGC: lateral gastrocnemius; asterisk: anterior bundle of the short head. **Supplemental Fig. 4.** Sonographic imaging (long-axis view) reveals the plantaris muscle at its proximal level (A), middle portion (B), distal level (C), plantaris tendon (asterisk) beneath the medial gastrocnemius (D), origin of the plantaris (E), and the muscle–tendon junction (F). CPN: common peroneal nerve; TN: tibial nerve; BIL: long head tendon of the biceps femoris; BIS: short head tendon of the biceps femoris; LGC: lateral gastrocnemius; MGC: medial gastrocnemius. **Supplemental Fig. 5.** Ultrasound imaging (short-axis view) of the arcuate ligament (asterisk) at the proximal side (A) and insertion (B). CPN: common peroneal nerve; BF: biceps femoris; LG: lateral gastrocnemius; PFL: popliteofibular ligament. **Supplemental Fig. 6.** Ultrasound imaging (long-axis view) reveals the normal (white arrowhead) (A) and sprained (black arrow) (B) popliteofibular ligaments. **Supplemental Fig. 7.** Ultrasound (A) and magnetic resonance imaging (B) show a ganglion cyst (white arrows) next to the popliteus. Ultrasound-guided aspiration (C) and the jelly-like content (D) of the cyst. BIF: biceps femoris; black arrows: needle.

**Additional file 2:** **Supplemental Video 1.** Two hypoechoic circles appearing deep to the fat tissue.

**Additional file 3:** **Supplemental Video 2.** Before the biceps femoris finally attaches to the fibular head, an anterior bundle emerges from the short head to insert on the tibial plateau.

**Additional file 4:** **Supplemental Video 3.** The case of a sprained lateral collateral ligament.

**Additional file 5:** **Supplemental Video 4.** A certain normal variations occurring where a bifid lateral collateral ligament may envelop the tendon itself.

**Additional file 6:** **Supplemental Video 5.** Conducting dynamic scanning of the arcuate ligament in the short-axis view.

**Additional file 7:** **Supplemental Video 6.** Aspiration of the popliteal tendon cyst.

## Data Availability

The datasets used and/or analyzed during the current study are available from the corresponding author on reasonable request.

## Abbreviations

LCL: Lateral collateral ligament
MR: Magnetic resonance
