# Supplementary material for: Ultrasound imaging of the posterior lateral corner of the knee: a pictorial review of anatomy and pathologies
Source: Insights Imaging. 2024 Feb 9;15:39. doi: 10.1186/s13244-024-01606-x (PMC10857999; doi:10.1186/s13244-024-01606-x)
Supplement: Supplementary file 1 — Additional file 1: Checklist of the PLC of the Knee. Supplemental Fig. 1. Magnetic resonance imaging shows the posterior lateral corner of the knee in the axial plane at the tibiofibular joint level. Supplemental Fig. 2. Sonographic imaging shows the posterior lateral corner of the knee in the transverse plane (A). Corresponding ultrasound images (long-axis view) of the iliotibial band (B), the anterolateral ligament (C), and the biceps femoris tendon (D). ITB: iliotibial band; ALL: anterolateral ligament; LCL: lateral collateral ligament; LM: lateral meniscus; BIL: long head tendon of the biceps femoris; BIS: short head tendon of the biceps femoris. Supplemental Fig. 3. Sonographic imaging shows the biceps femoris in the transverse plane; the myotendinous junction (A), anterior bundle from the short head (B), its attachment to the fibular head (C). Imaging in long-axis view (D). LCL: lateral collateral ligament; BIL: long head tendon of the biceps femoris; BIS: short head tendon of the biceps femoris; LGC: lateral gastrocnemius; asterisk: anterior bundle of the short head. Supplemental Fig. 4. Sonographic imaging (long-axis view) reveals the plantaris muscle at its proximal level (A), middle portion (B), distal level (C), plantaris tendon (asterisk) beneath the medial gastrocnemius (D), origin of the plantaris (E), and the muscle–tendon junction (F). CPN: common peroneal nerve; TN: tibial nerve; BIL: long head tendon of the biceps femoris; BIS: short head tendon of the biceps femoris; LGC: lateral gastrocnemius; MGC: medial gastrocnemius. Supplemental Fig. 5. Ultrasound imaging (short-axis view) of the arcuate ligament (asterisk) at the proximal side (A) and insertion (B). CPN: common peroneal nerve; BF: biceps femoris; LG: lateral gastrocnemius; PFL: popliteofibular ligament. Supplemental Fig. 6. Ultrasound imaging (long-axis view) reveals the normal (white arrowhead) (A) and sprained (black arrow) (B) popliteofibular ligaments. Supplemental Fig. 7. Ultrasound (A) [file 13244_2024_1606_MOESM1_ESM.docx]

**Ultrasound Imaging of the Posterior Lateral Corner of the Knee: A Pictorial Review of Anatomy and Pathologies**

**ELECTRONIC SUPPLEMENTARY MATERIAL**

**Checklist of the PLC of the Knee**

|  | **Adequate image**  **obtained?** | | **Structure**  **identified?** | | **Pathology** | |
| --- | --- | --- | --- | --- | --- | --- |
|  | **Yes** | **No** | **Yes** | **No** | ***Yes** | **No** |
| **Iliotibial band**  **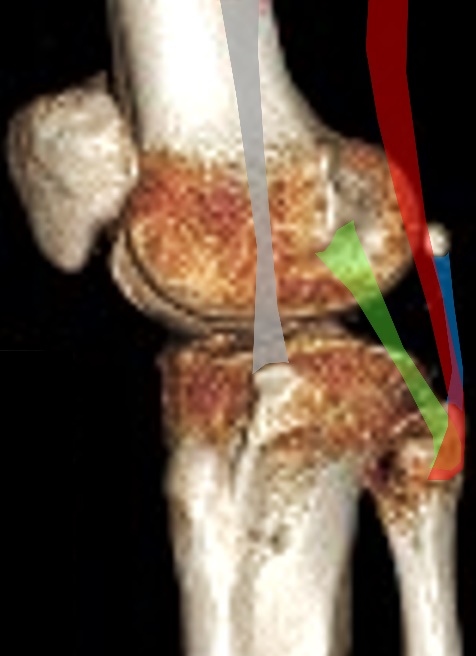** |  |  |  |  |  |  |
| **Anterolateral ligament**  **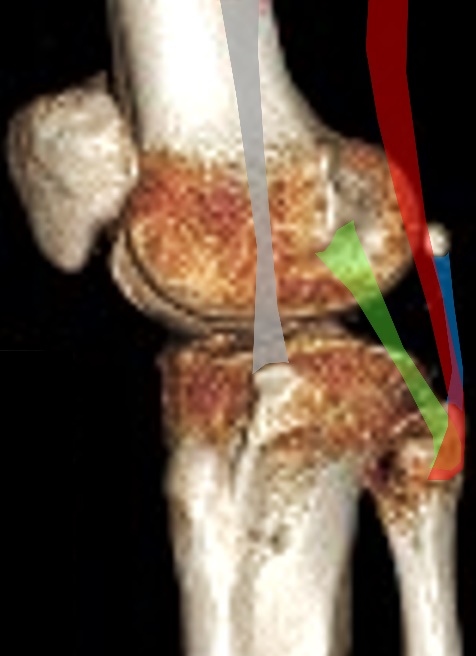** |  |  |  |  |  |  |
| **Lateral collateral ligament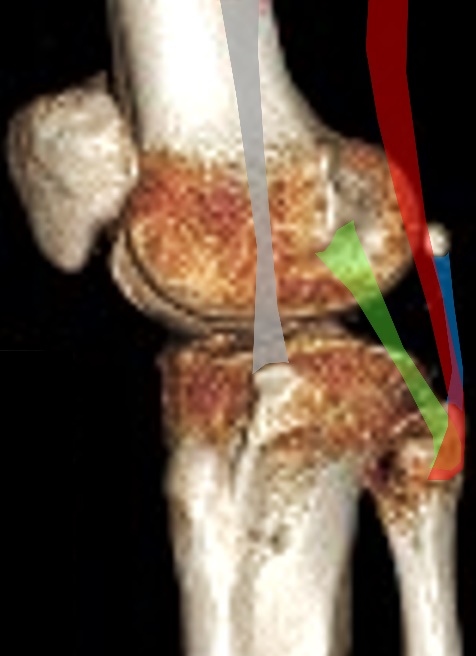** |  |  |  |  |  |  |
| **Popliteus tendon/muscle**  **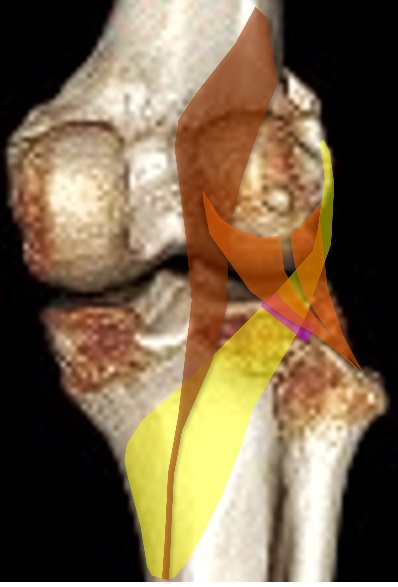** |  |  |  |  |  |  |
| **Popliteofibular ligament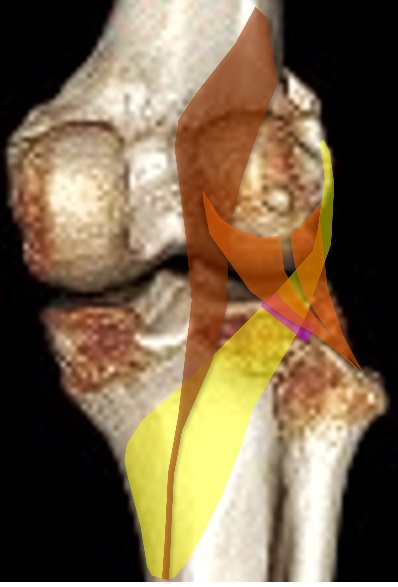** |  |  |  |  |  |  |
| **Arcuate ligament**  **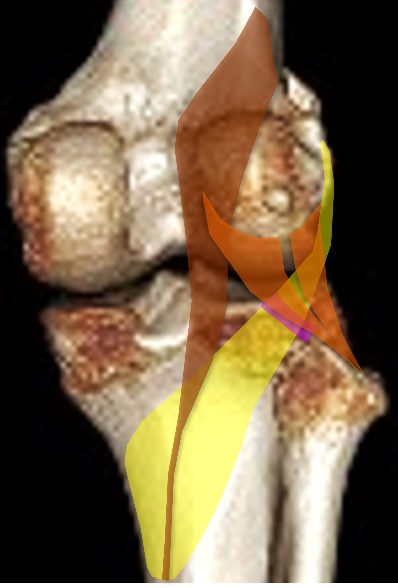** |  |  |  |  |  |  |
| **Biceps femoris tendon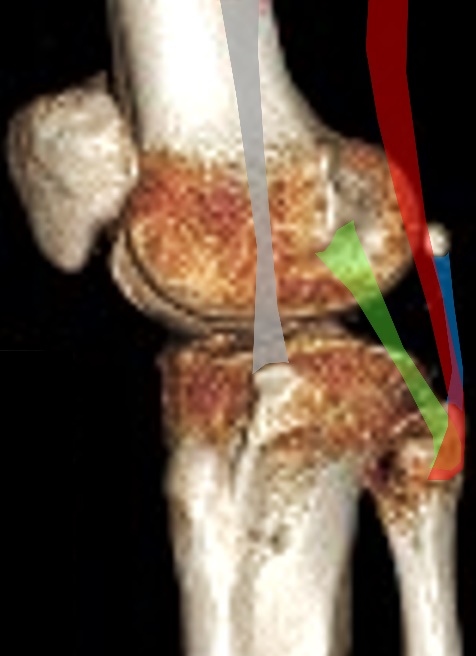** |  |  |  |  |  |  |
| **Fabellofibular ligament**  **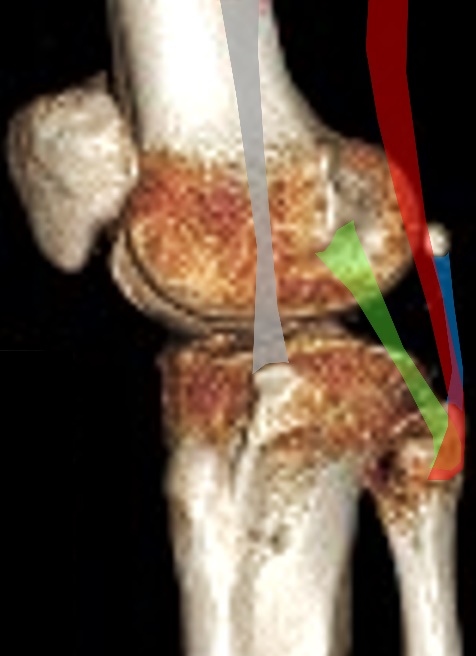** |  |  |  |  |  |  |
| **Plantaris muscle/tendon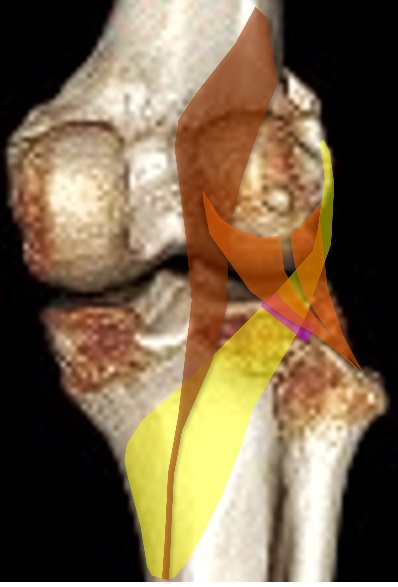** |  |  |  |  |  |  |


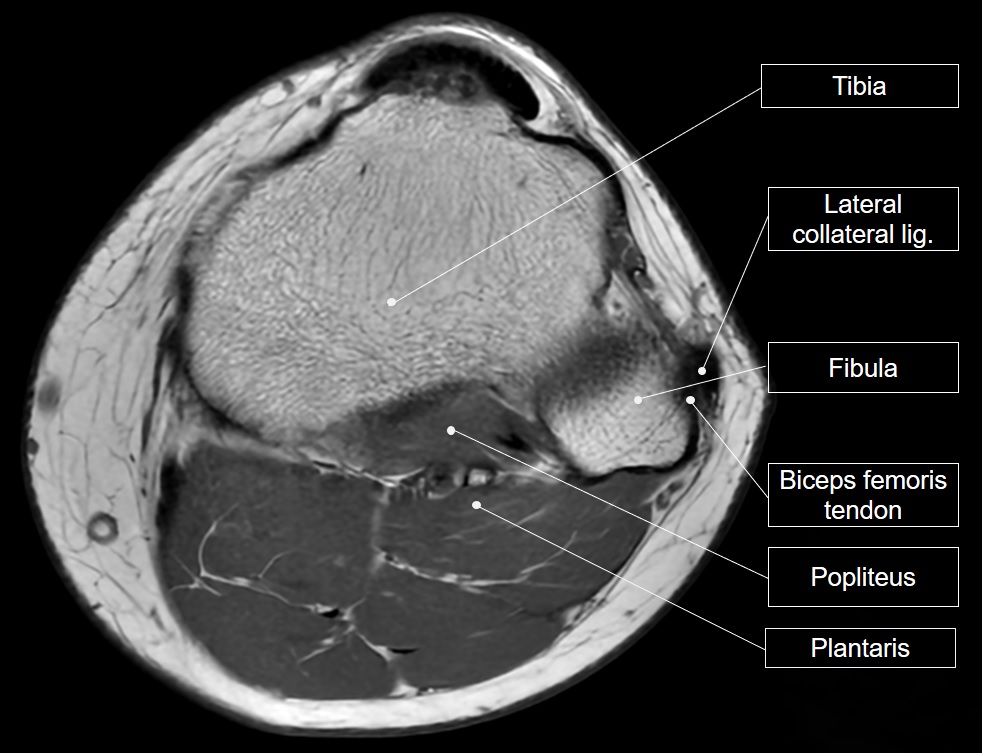


**Supplemental Figure 1** Magnetic resonance imaging shows the posterior lateral corner of the knee in the axial plane at the tibiofibular joint level.


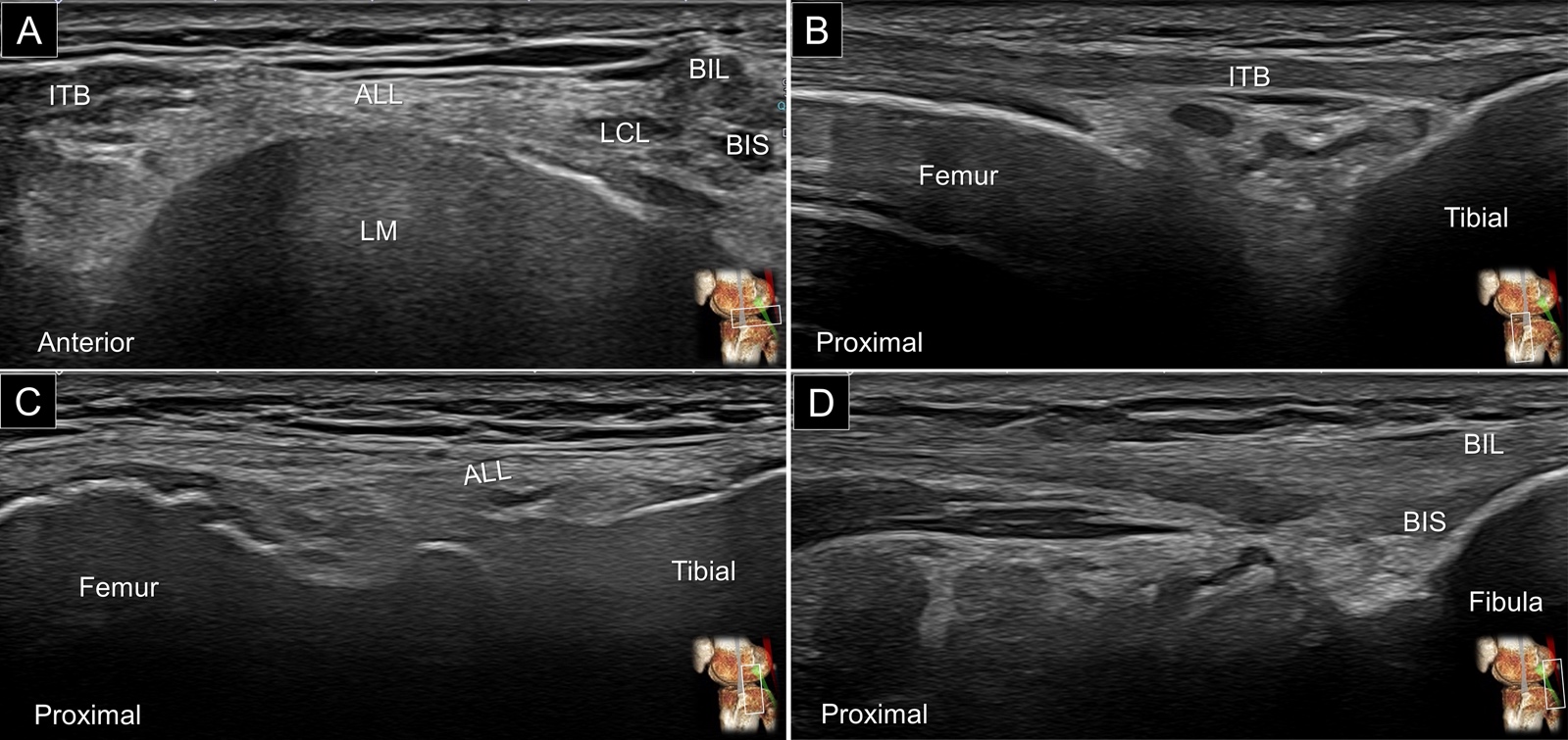


**Supplemental Figure 2** Sonographic imaging shows the posterior lateral corner of the knee in the transverse plane **(A)**. Corresponding ultrasound images (long-axis view) of the iliotibial band **(B)**, the anterolateral ligament **(C)**, and the biceps femoris tendon **(D)**.

ITB: iliotibial band; ALL: anterolateral ligament; LCL: lateral collateral ligament; LM: lateral meniscus; BIL: long head tendon of the biceps femoris; BIS: short head tendon of the biceps femoris.


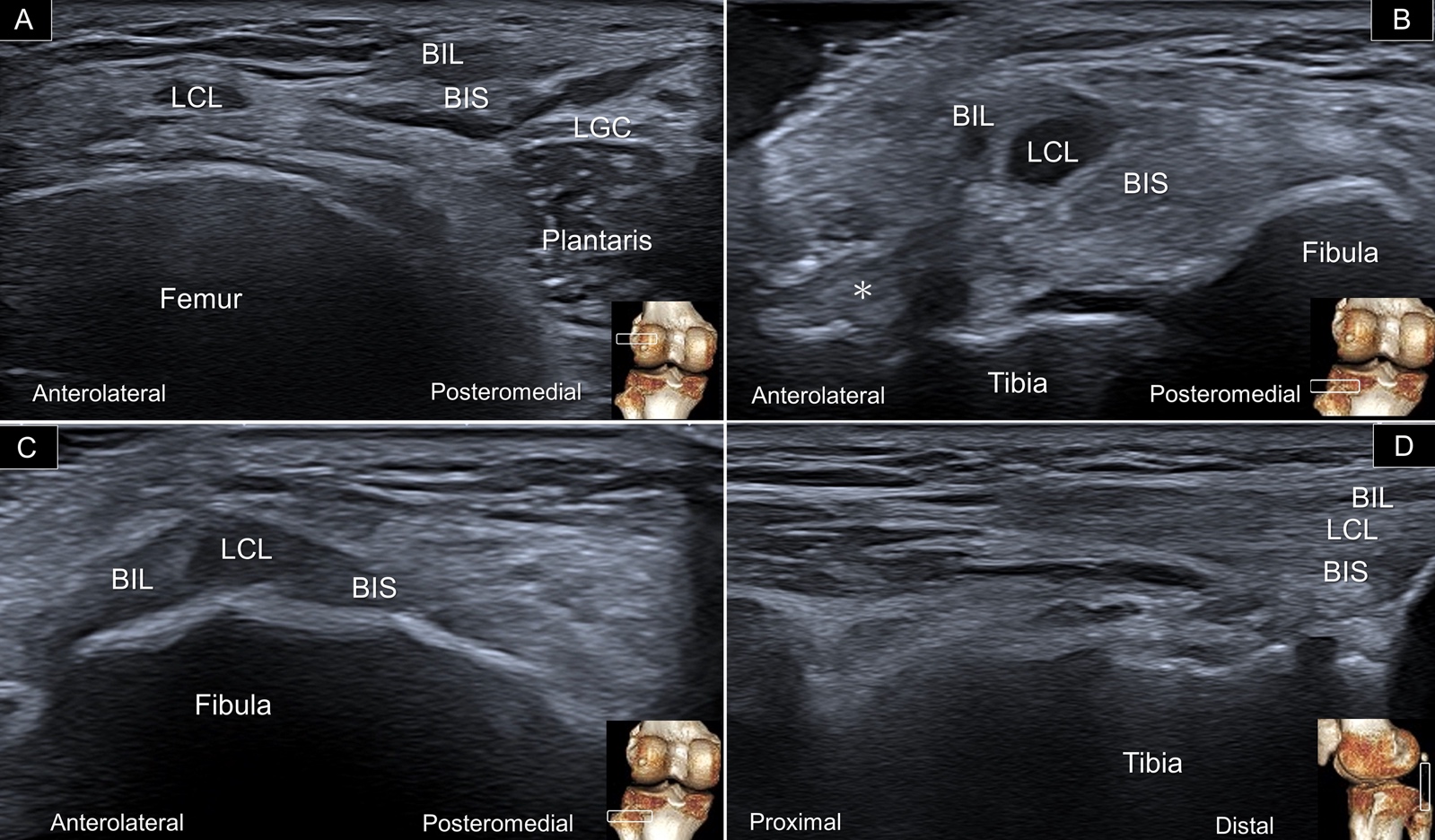


**Supplemental Figure 3** Sonographic imaging shows the biceps femoris in the transverse plane; the myotendinous junction **(A)**, anterior bundle from the short head **(B)**, its attachment to the fibular head **(C)**. Imaging in long-axis view **(D)**.

LCL: lateral collateral ligament; BIL: long head tendon of the biceps femoris; BIS: short head tendon of the biceps femoris; LGC: lateral gastrocnemius; asterisk: anterior bundle of the short head.


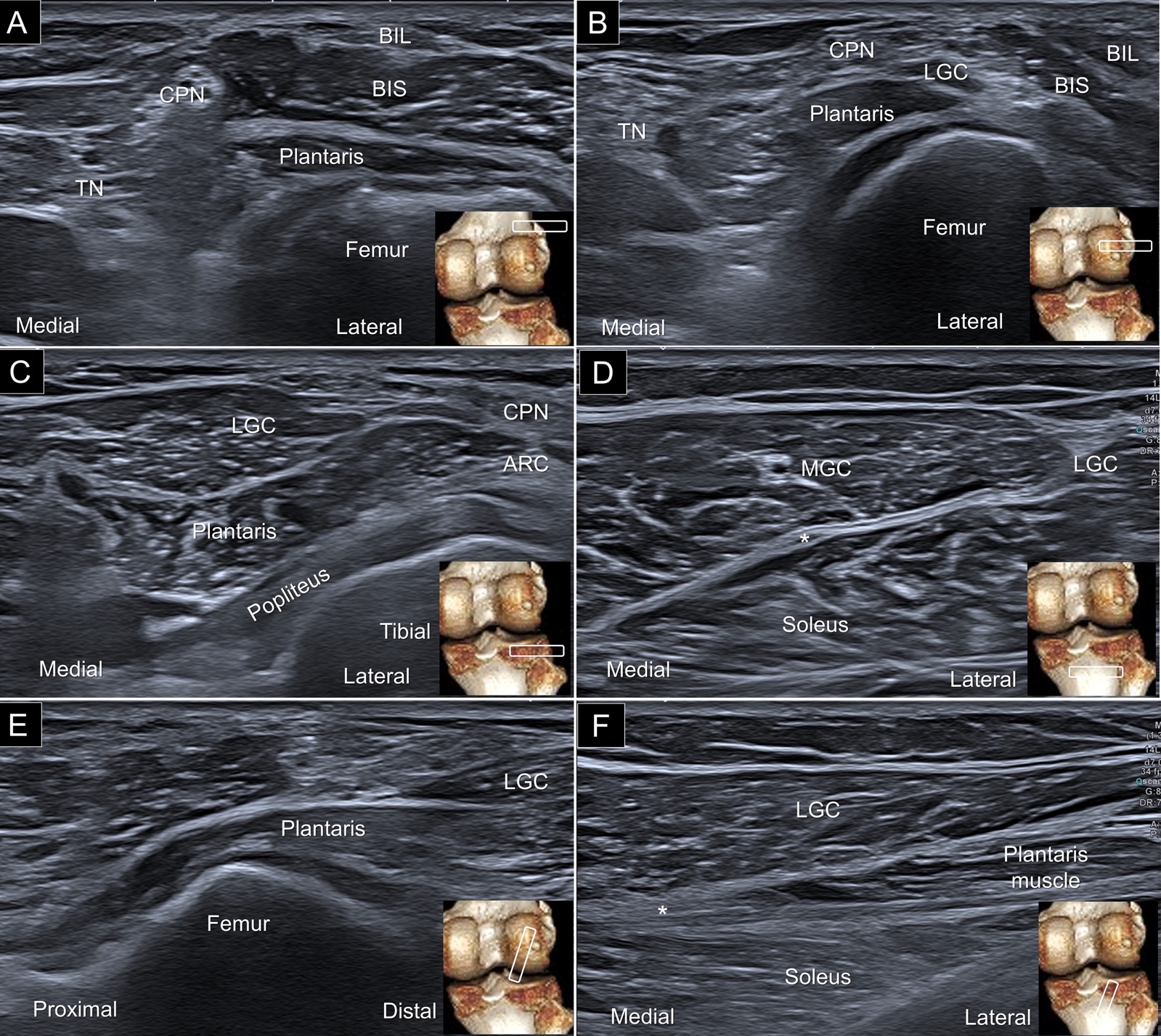


**Supplemental Figure 4** Sonographic imaging (long-axis view) reveals the plantaris muscle at its proximal level **(A)**, middle portion **(B)**, distal level **(C)**, plantaris tendon (asterisk) beneath the medial gastrocnemius **(D)**, origin of the plantaris **(E)**, and the muscle-tendon junction **(F)**.

CPN: common peroneal nerve; TN: tibial nerve; BIL: long head tendon of the biceps femoris; BIS: short head tendon of the biceps femoris; LGC: lateral gastrocnemius; MGC: medial gastrocnemius.


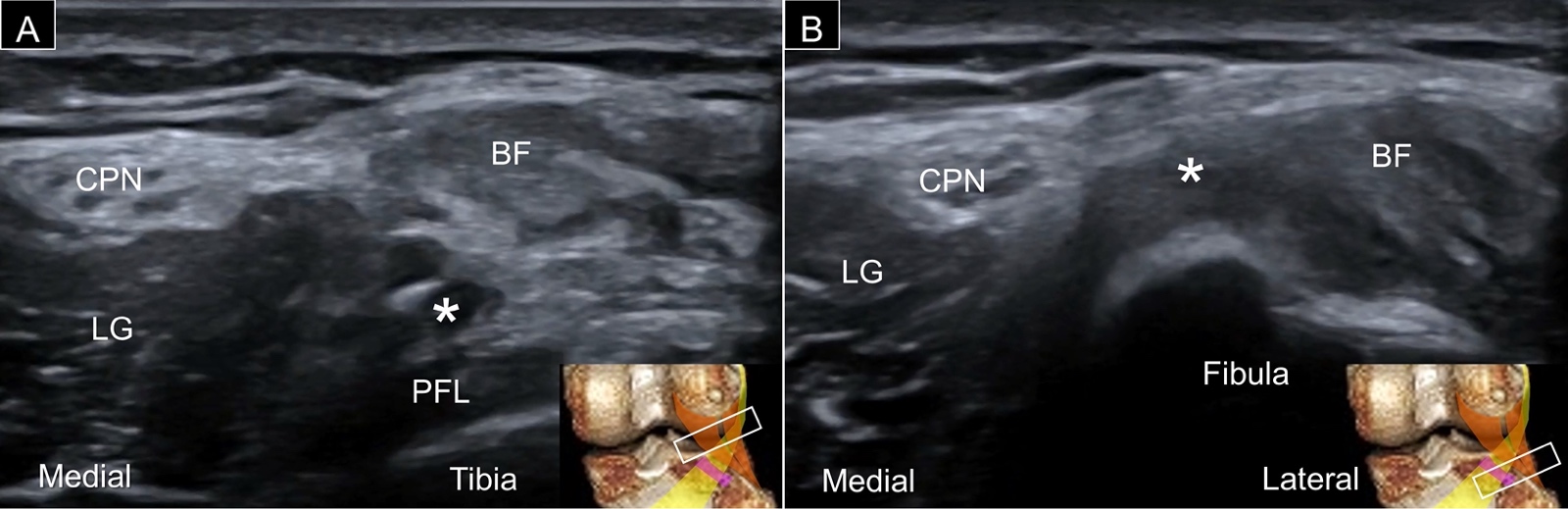


**Supplemental Figure 5** Ultrasound imaging (short-axis view) of the arcuate ligament (asterisk) at the proximal side **(A)** and insertion **(B)**.

CPN: common peroneal nerve; BF: biceps femoris; LG: lateral gastrocnemius; PFL: popliteofibular ligament.


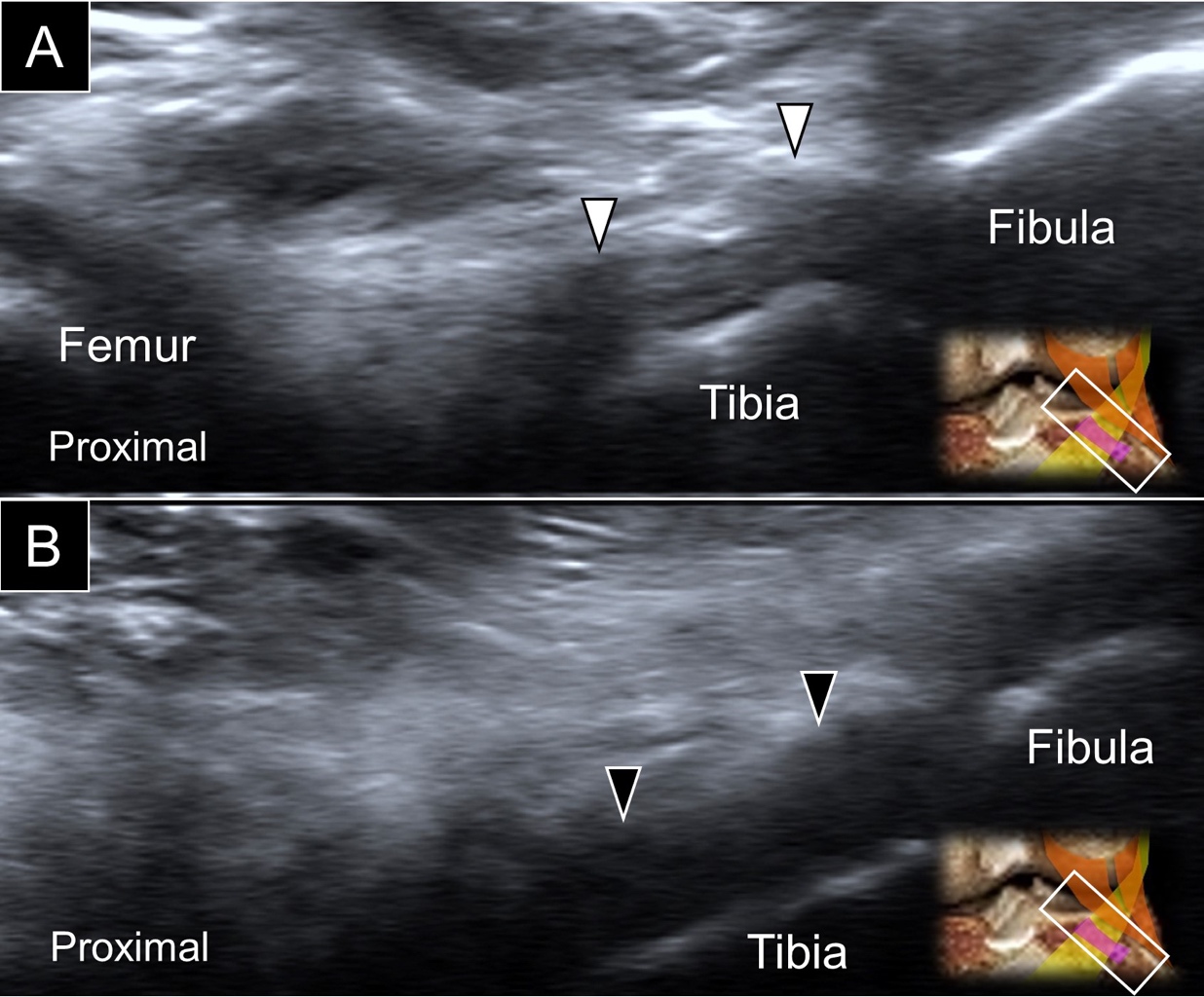


**Supplemental Figure 6** Ultrasound imaging (long-axis view) reveals the normal (white arrowhead) **(A)** and sprained (black arrow) **(B)** popliteofibular ligaments.


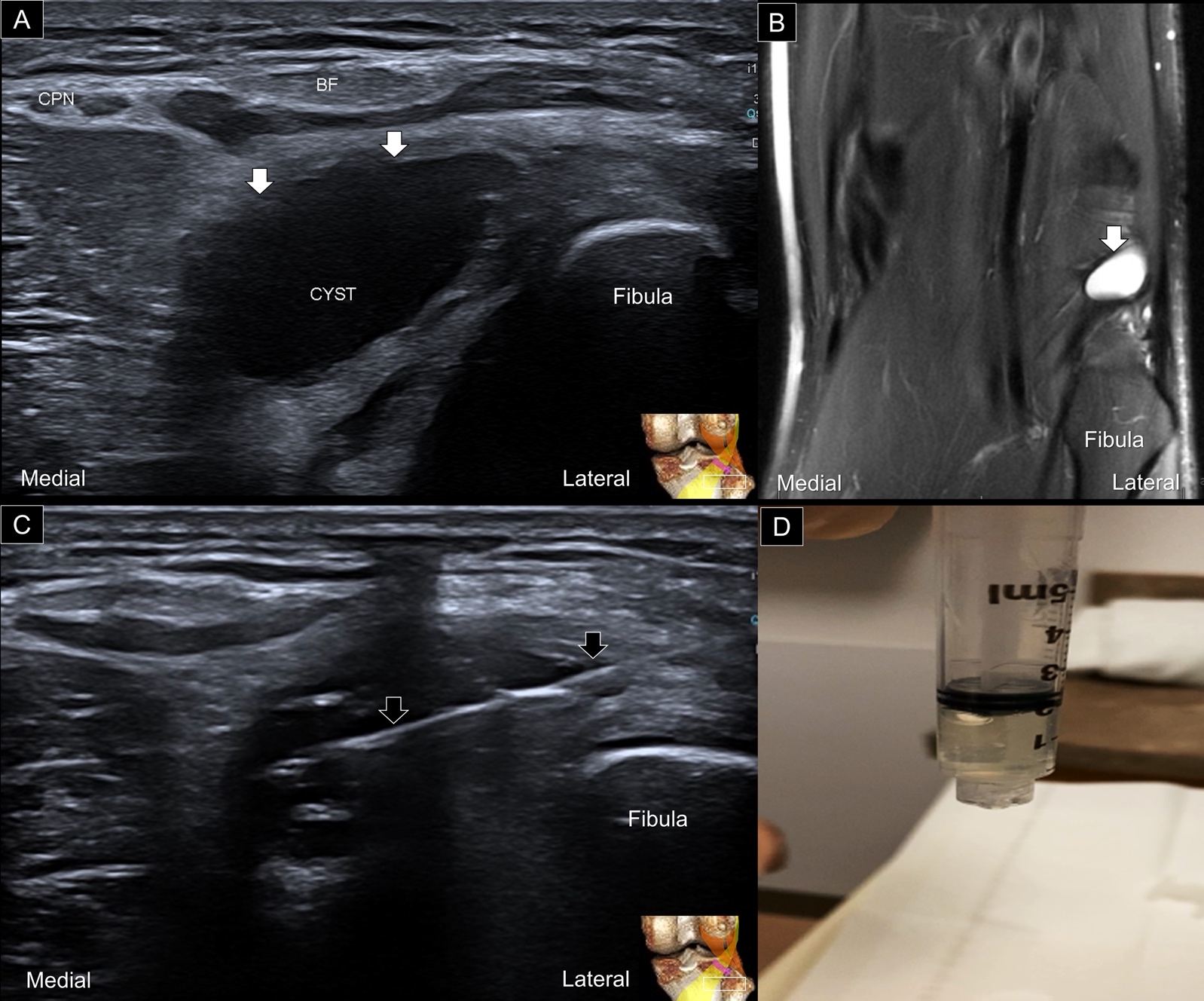


**Supplemental Figure 7** Ultrasound **(A)** and magnetic resonance imaging **(B)** show a ganglion cyst (white arrows) next to the popliteus. Ultrasound-guided aspiration **(C)** and the jelly-like content **(D)** of the cyst.

BIF: biceps femoris; black arrows: needle.
